# The BioFilm Ring Test: a Rapid Method for Routine Analysis of Pseudomonas aeruginosa Biofilm Formation Kinetics

**DOI:** 10.1128/JCM.02938-15

**Published:** 2016-02-25

**Authors:** Elodie Olivares, Stéphanie Badel-Berchoux, Christian Provot, Benoît Jaulhac, Gilles Prévost, Thierry Bernardi, François Jehl

**Affiliations:** a Fédération de médecine translationnelle, EA7290 Virulence bactérienne précoce, Institut de Bactériologie, Université de Strasbourg, Strasbourg, France; b BioFilm Control SAS, Saint-Beauzire, France

## Abstract

Currently, few techniques are available for the evaluation of bacterial biofilm adhesion. These detection tools generally require time for culture and/or arduous handling steps. In this work, the BioFilm Ring Test (BRT), a new technology, was used to estimate the biofilm formation kinetics of 25 strains of Pseudomonas aeruginosa, isolated from the sputum of cystic fibrosis (CF) patients. The principle of the new assay is based on the mobility measurement of magnetic microbeads mixed with a bacterial suspension in a polystyrene microplate. If free to move under the magnetic action, particles gather to a visible central spot in the well bottom. Therefore, the absence of spot formation in the plate reflects the bead immobilization by a biofilm in formation. The BRT device allowed us to classify the bacterial strains into three general adhesion profiles. Group 1 consists of bacteria, which are able to form a solid biofilm in <2 h. Group 2 comprises the strains that progressively set up a biofilm during 24 h. Lastly, group 3 includes the strains that stay in a planktonic form. The grouping of our strains did not differ according to culture conditions, i.e., the use of different sets of beads or culture media. The BRT is shown to be an informative tool for the characterization of biofilm-forming bacteria. Various application perspectives may be investigated for this device, such as the addition of antibiotics to the bacterial suspension to select which would have the ability to inhibit the biofilm formation.

## INTRODUCTION

Pseudomonas aeruginosa, a Gram-negative rod-shaped bacterium, is an ubiquitous microorganism widespread in different environments, such as soil, water, plants, animals, and humans. This opportunistic pathogen often colonizes immunocompromised patients. It is largely involved in hospital-acquired infections, including pneumonia, burn, wound, urinary tract and gastrointestinal infections, otitis media, and keratitis (1). P. aeruginosa infections occur frequently in cystic fibrosis (CF) patients, and the presence of bacteria in airways is highly associated with poor lung function, morbidity, and mortality (2, 3). Once in CF lungs, P. aeruginosa is virtually impossible to eradicate, and despite the inflammatory response and intensive antibiotic treatments, infections caused by this pathogen persist. In fact, bacteria are able to survive in stressful environments by switching to the biofilm mode of growth (4).

Biofilms are structured communities of bacteria embedded in a self-produced matrix composed of exopolysaccharides, proteins, and extracellular DNA. Bacterial biofilms are notoriously known for their high resistance to antibiotics, disinfectant chemicals, and components of the innate and adaptive inflammatory defense system of the body (5).

Antibiotic tolerance in biofilms is 10- to 1,000-fold higher than in corresponding planktonic bacteria (6). Biofilm-reduced susceptibility to antibiotics arises from the combination of several mechanisms, including slow antibiotic penetration in the biofilm matrix, slow bacterial growth in an altered microenvironment (nutrient gradients and oxygen restriction), resort of quorum-sensing mechanisms by bacteria, and existence of a population of persister microorganisms (7, 8).

Several methods are available to measure bacterial biofilm adherence and to test biofilm susceptibility to antimicrobial agents (9). For the numeration of sessile bacteria after their surface detachment, culture (colony formation) and staining methods can be used, in addition to quantitative PCR (qPCR) and various microscopy techniques, such as epifluorescence and laser-scanning confocal, transmission electron, and scanning electron microscopy (10–16).

A new technology called the BioFilm Ring Test (BRT) (BioFilm Control, Saint-Beauzire, France) was developed. The assay does not require any washing or staining steps, it is easy to handle, and above all, results can be obtained in a few hours. Briefly, a bacterial suspension is mixed with superparamagnetic microbeads. If a biofilm is forming, microparticles are embedded in the matrix and, after magnetization, are no longer detectable. Based on the measurement of this superparamagnetic microbead immobilization by adherent cells, the BRT can be used to assess the kinetics of biofilm formation and the ability of antibiotics to prevent it (17, 18).

Here, we used the device for the evaluation of bacterial biofilm formation by a collection of P. aeruginosa strains isolated from sputum samples of CF patients.

## MATERIALS AND METHODS

### Bacterial strains and growth conditions.

Twenty-five strains of P. aeruginosa isolated from the sputum of a cohort of CF patients were analyzed in this study. All strains were collected from patients of the CF Foundation (Centre de Ressources et de Compétences de la Mucoviscidose) of the University Hospital of Strasbourg (France).

They were identified by mass spectrometry by using the matrix-assisted laser desorption ionization (MALDI) Biotyper. Frozen cultures were then prepared in brain heart infusion (BHI) broth, supplemented by 10% (vol/vol) of glycerol, and stored at −80°C for further use. When experiments were planned, loopfuls of these frozen cultures were defrosted, spread on Drigalski agar plates, and incubated at 37°C for 24 h. From these subcultures, strains were maintained weekly on Drigalski agar plates, from one colony of the previous culture.

When an adhesion kinetic test was scheduled, strains were precultured the day before the experiment on a BHI or Müeller-Hinton (MH) agar plate.

### Preparation of initial bacterial suspension.

For each of the 25 bacterial strains included in the study, adhesion kinetic experiments were carried out with two culture media. The BHI medium is recommended by the manufacturer for BRT use, but the MH medium was also tested as it is officially recommended by the European Committee on Antimicrobial Susceptibility Testing (EUCAST) for antimicrobial susceptibility testing on bacteria (19). Thus, some colonies from agar cultures were cautiously resuspended in 2 ml of sterile liquid medium. These solutions were employed to bring initial bacterial suspension (IBS) cultures to a final optical density adjusted to 1/250 (4 × 10^6^ CFU/ml) at 600 nm (OD_600_).IBS was then used for kinetic tests carried out on the BRT device.

### Bacterial adhesion assessment using BRT.

Each of the 25 P. aeruginosa strains was tested to evaluate its adhesion capacity to polystyrene 96-well microplates using the BRT. To carry out these tests, microplates (12 columns of 8 wells), toner solution (containing magnetic microbeads), contrast liquid (an inert opaque oil used for the reading step), a block test (the magnet support), and the dedicated scan plate reader (a commercial Epson scanner modified for microplate reading) were used.

The toner solution was mixed for homogenization and added to each IBS to get a final concentration of 10 μl · ml^−1^. Two sets of beads (toner 4 and toner 6) are available to test their impact on the biofilm formation ability of strains. They mainly differ in diameter, resulting in a variation in their sedimentation speed; therefore, this mixture was homogenized by vortexing, and 200 μl were loaded per well. One column was used for each P. aeruginosa strain tested (11 strains can be tested on a plate), and the last one was used as a control for bead migration (culture broth and toner solution without bacteria). Five plates were prepared for each incubation time tested (0, 2, 4, 6, and 24 h). The cultures were incubated at 37°C.

After incubation, wells were covered with ∼120 μl of contrast liquid. Plates were read before (I_0_ image getting) and after (I_1_ image getting) 1 min of magnetization using the block test and the scanner. The block test is made up of 96 minimagnets, which are centered under the bottom of each well. After magnet contact, free beads are attracted toward the center of the wells, forming a brown spot, while beads embedded in a biofilm are blocked and remain undetectable. The adhesion ability of each strain was expressed as the biofilm index (BFI) according to dedicated software, the BioFilm Control Elements. The software compares the I_0_ and I_1_ images of each well and, through a mathematical algorithm, calculates a corresponding BFI value ranging from 0 to 30. Deduced BFIs are inversely proportional to the attached cell number. A high BFI value indicates high bead mobility under magnetic action that corresponds to the absence of biofilm formation (i.e., control wells), while a low value (∼2) shows a complete immobilization of beads due to the sessile cells (no difference between well images I_0_ and I_1_).

Three independent experiments, with at least four replicates (four wells) by experiment for each strain and condition tested (toner sets and culture media), were carried out. Two-way analysis of variance (ANOVA) was performed on the resulting BFI data.

## RESULTS

### Tests of adhesion kinetics.

For each test, adherence was measured at 0, 2, 4, 6, and 24 h. Three groups of strains emerged. The first group included the strains that rapidly form a biofilm (in <2 h). The second group included strains that form a biofilm slowly and progressively. The third group comprised strains that were not able to form a biofilm.

**FIG 1:**
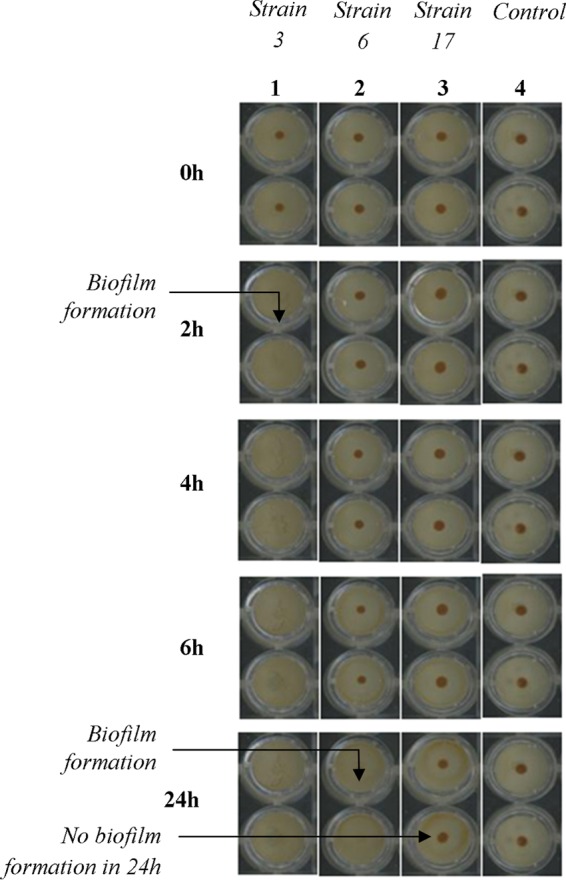
Kinetics of biofilm formation by three selected P. aeruginosa strains with the BioFilm Ring Test at different incubation times. Strip 4, controls with BHI medium and toner alone. Well images were obtained by scan of microplates with the plate reader after magnetization by the block test.

The kinetic profiles for three strains representative of these three groups (P. aeruginosa 3, 6, and 17, respectively) are presented in Fig. 1. Testing of controls consisting of sterile BHI medium with toner was performed to show that beads were freely mobile in the absence of sessile cells. All control wells, at any incubation times, were characterized by a centered brown spot after plate magnetization (Fig. 1, strip 4). This corresponds to the attraction and concentration of beads under magnetic action. Thus, shape or intensity variations of the spots in wells containing bacteria may be attributed only to the presence of microorganisms. For strain 3, spots visualized in the initial plate (0 h) had completely disappeared after 2 h incubation (Fig. 1, strip 1). For strain 6, brown spots were observed only at the beginning of the experiment (Fig. 1, strip 2). Due to the progressive adhesion of bacterial cells, the magnetic forces were not strong enough to attract beads at the well bottom or to overcome their trapping during biofilm formation. Thus, the spot intensity was reduced during the time experiment until its disappearance. Finally, the biofilm-negative strain 17 displayed brown spots throughout the kinetic analysis, showing that beads were always mobile under magnetic action and were not retained by potential sessile cells (Fig. 1, strip 3).

**FIG 2:**
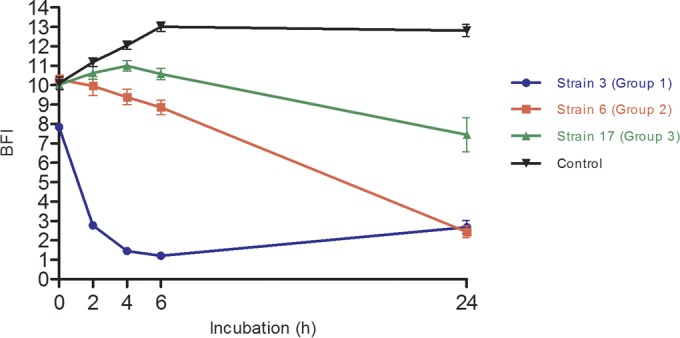
Biofilm formation by P. aeruginosa strains 3, 6, and 17 obtained after analysis of microplate images by the BioFilm Control Elements software. Control curve represents the BHI medium with toner condition. Results are expressed as BFI (biofilm index) as a function of incubation time. Analyses of results obtained by two-way ANOVA validated a significant difference between each point of the different strain curves. Bars, standard deviation obtained from 12 measurements obtained for each strain (3 independent experiments performed in quadruplicate).

The raw data interpretation of biofilm formation kinetics is shown in Fig. 2. As previously described, the plate images analyzed by the software gives a BFI value for each strain at each incubation point. When displayed as a function of time, this index gives a kinetic profile in concordance with the bead attraction during the magnetization step, reflecting the biofilm formation. Thus, a high BFI value (>8) reflects complete bead mobility corresponding to the absence of a biofilm, whereas a low value (≤2) reflects the complete immobilization of beads (embedded in the formed biofilm). The biofilm formation kinetics of the depicted strains differentiated three adhesion behaviors. Strain 3 formed a strong biofilm in <2 h incubation (BFI <3 at 2 h), and this biofilm persisted until the end of the kinetic measurement. Strain 6 displayed a progressive decrease in BFI values, reflecting slower but constant biofilm formation. Finally, for strain 17, BFI values remained at >8 throughout the experiment, showing that this strain was not able to adhere to the microplates. Bacteria grew in suspension in the medium in a planktonic state.

All of the P. aeruginosa strains of the collection, originating from CF patients, were classified in one of these three adhesion profiles. Among the 25 strains tested, 7 formed a strong biofilm in <2 h (adhesion profile 1), only 4 strains of the collection did not form a biofilm after 24 h incubation (adhesion profile 3), and more than half (14) of the collection strains formed a biofilm in a progressive manner during 24 h (adhesion profile 2) (data not shown).

Moreover, the good reproducibility of three independent experiments showed that these adhesion profiles were a conserved feature by each strain. Standard deviations on graphs were representative, for each analysis point, of 12 BFI measurements (four replicates by test).

### Effects of the toner solution on biofilm formation.

**FIG 3:**
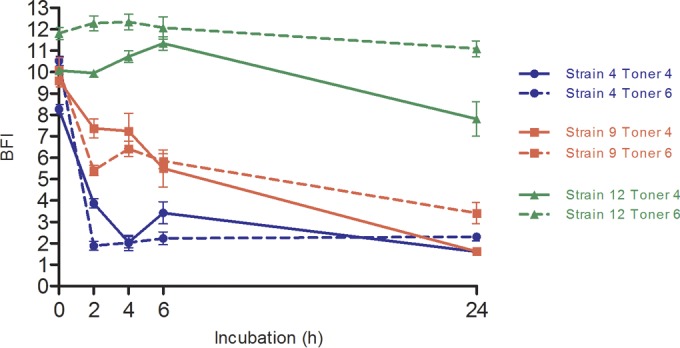
Adhesion kinetics of P. aeruginosa strains 4, 9, and 12 with two sets of paramagnetic microbeads (toner 4 and toner 6). Results were obtained after analysis of microplate images by the BioFilm Control Elements software and are expressed as BFI (biofilm index) as a function of incubation time. Analyses of results obtained by two-way ANOVA validated a statistically significant difference between each point of the different strain curves. Conversely, changing microbead sets did not implicate such significant differences between toner curves for the same strain. Bars, standard deviation obtained from 12 measurements obtained for each strain (3 independent experiments performed in quadruplicate).

Another set of microbeads (toner 6) was tested in regard to the adhesion kinetics of the 25 P. aeruginosa strains. This toner solution differed in bead diameter from the one used initially (toner 4). Results for strains 4, 9, and 12, belonging to adhesion profiles 1, 2, and 3, respectively, are shown in Fig. 3.

Mostly, the size of beads did not interfere in the adherence ability of bacteria. No significant differences were revealed between experiments carried out with the two toner solutions. Indeed, for a given strain, the two adhesion curves were nearly superimposed (Fig. 3). This concordance between the two toner solutions was observed for all 25 P. aeruginosa strains (data not shown).

### Effects of the culture medium on biofilm formation.

**FIG 4:**
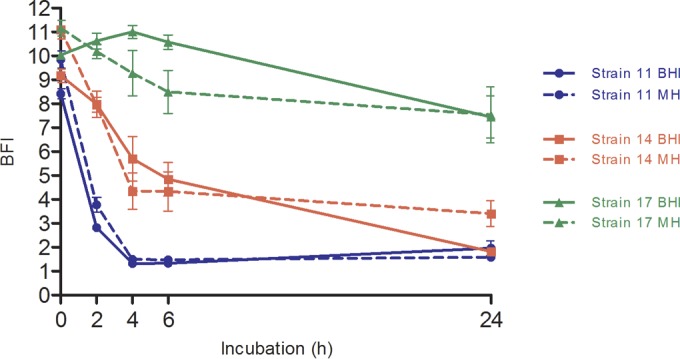
Biofilm formation kinetics of P. aeruginosa strains 11, 14, and 17 in two culture media (BHI and MH). Results were obtained after analysis of microplate images by the Biofilm Control Elements software and are expressed as BFI (biofilm index) as a function of incubation time. Analyses of results obtained by two-way ANOVA validated a statistically significant difference between each point of the different strain curves. Conversely, changing media did not implicate such significant differences between the two different curves for the same strain. Bars, standard deviation obtained from 12 measurements obtained for each strain (3 independent experiments performed in quadruplicate).

The BHI medium is theoretically recommended by the manufacturer for experiments carried out with the BRT device. The MH medium, which is officially recommended by EUCAST for the realization of antimicrobial susceptibility testing on bacteria, was used for comparison (19). An example of results obtained with strains 11, 14, and 17 (belonging to adhesion profiles 1, 2, and 3, respectively) is shown in Fig. 4. These culture media did not influence the bacteria adhesion capacity. These concordant behaviors between the media were found for the whole P. aeruginosa collection (data not shown). Therefore, results obtained using BHI medium can be correlated with those obtained using MH.

## DISCUSSION

Currently, a limited number of methods are available to detect biofilm formation by bacteria. The conventional methods are usually quantification by staining (e.g., crystal violet [CV] test) or observation by microscopy (9–14). However, staining is a tedious technique with washing steps that may lead to the detachment and loss of sessile bacteria. Similarly, the use of microscopy techniques is arduous because it requires delicate steps for sample preparation. This explains why these standard methods are not adapted for routine use.

In our hands, the BRT device allowed the classification of P. aeruginosa strains in three profiles according to their adhesion ability. Comparison with the CV assay was done with eight replicates for each strain at each sampling time. The adhesion profiles detected by the CV method were exactly the same as those detected by the BRT (data not shown).

Moreover, in order to assess the performance and reliability of the BRT, experiments of adhesion kinetics were performed three times for each strain with four replicates by test. Note that use of the BRT device requires very few manipulations aside from the initial bacterial suspension preparation. Therefore, the very low standard deviation obtained and the features, including less time required and less handling compared to the standard methods, are key arguments for device certification.

Finally, as mentioned by Chavant et al. (17), it is important to test various conditions to confirm the robustness of this new technology. Results obtained with another culture medium (MH) and another microbead set (toner 6) led to similar trends concerning the adhesion ability of the strains, strengthening the predictability of the BRT.

Many perspectives of utilization of this methodology are under investigation in our laboratory. The addition of various antibiotics to wells to perform an Antibiofilmogram test may enable detection of the ability of a given molecule to inhibit or delay biofilm formation. Indeed, Antibiofilmograms may soon be used for antimicrobial susceptibility testing in diagnostic laboratories to complete data obtained from conventional antibiograms (20). These experiments are under way. First trends confirm that it may be possible to find antibiotics that are inhibitors of biofilm formation in a strain-dependent manner.

Another potential use of the BRT is for detection of the inducer effects of some antibiotics on the setting up of biofilm by a strain that initially does not form one. The clinical impact of such results would be considerable if they enable discarding the use of inappropriate antibiotic treatments that would lead to biofilm formation in CF patients. Owing to the fact that P. aeruginosa in a sessile mode in lungs is responsible for the general deterioration of patients, it seems very important to confirm and study these observations in more detail.

Other experiments are planned to determine whether the acquisition of the biofilm formation phenotype by bacteria following antibiotic treatment is an irreversible phenomenon.

Macrolides are also known to reduce P. aeruginosa activity by acting on the quorum sensing or the bacterial mobility (21, 22). It may be interesting to evaluate the impact of this class of antibiotics, when added to any other class of antimicrobials, on the setting up of biofilm in our strains.

Finally, the results obtained here are specific to P. aeruginosa isolated from CF patients and cannot be extrapolated to other species. The use of rich media for cell growth does not reflect the natural environment of bacteria. Nevertheless, they are unavoidable since the usual clinical microbiological *in vitro* tests (classic antibiograms) are performed with rich medium (MH, BHI).

In conclusion, the BRT appears to be a relevant method for determination of the ability of P. aeruginosa to initiate the formation of a biofilm. In the near future, after certification, the device may be used to estimate antibiotic susceptibilities on sessile bacteria by carrying out Antibiofilmogram tests.
